# Factors affecting the maxillary and mandibular incisors’ buccolingual inclinations and buccal and lingual cortical plate heights

**DOI:** 10.1186/s12903-023-03225-2

**Published:** 2023-07-22

**Authors:** Seyed Mohammad Jafary Pour, Morteza Gooran, Arash Dabbaghi, Farnoush Parsi, Ali Rohani, Mehrnaz Moradinejad, Seyed Arman Mohagheghi, Vahid Rakhshan

**Affiliations:** 1 Department of Orthodontics, School of Dentistry, Ahvaz Jundishapur University of Medical Sciences, Ahvaz, Iran; 2 Department of Oral and Maxillofacial Radiology, School of Dentistry, Ahvaz Jundishapur University of Medical Sciences, Ahvaz, Iran; 3 Department of Dental Anatomy, Dental Faculty, Azad University of Medical Sciences, Tehran, Iran

**Keywords:** Alveolar bone height, Incisors’ buccolingual inclination, Orthodontics, Periodontics, Anatomy, Cone-Beam Computed Tomography

## Abstract

**Introduction:**

Orthodontics is closely related to periodontics. The buccolingual inclination (BLI) of the incisors and deficiencies in their buccal (BHd) and lingual (LHd) cortical plate heights may affect orthodontic outcomes. Identifying risk factors that can compromise buccal or lingual bone heights may have clinical value. The literature on BLI/BHd/LHd is not only scarce but also limited to one jaw. We aimed to examine, for the first time, factors affecting BLI/BHd/LHd not evaluated before as well as other factors with scarce literature about them.

**Methods:**

In this two-phase epidemiological and analytical study, inclinations and cortical heights of 248 incisors (bilateral centrals and laterals) were evaluated blindly on 62 randomly selected high-resolution pretreatment cone-beam computed tomography volumes (30 maxillae [13 men, 17 women], 32 mandibles [13 men, 19 women]). The sample was balanced in terms of sexes, jaws, and ages. The BLI/BHd/LHd of bilateral incisors were measured (intraobserver agreement > 95%). The effects of jaws, sexes, age, sides, and incisor types on each of the anatomical variables (BLI/BHd/LHd) were analyzed using a Mixed-Model Multiple Linear Regression analysis. Correlations among continuous variables were assessed using a Pearson coefficient (α = 0.05).

**Results:**

For the maxillary centrals, BLI, BHd, and LHd were 106.79 ± 5.06, 1.94 ± 0.95, and 1.50 ± 0.76, respectively. These parameters were ‘110.56 ± 5.97, 1.81 ± 0.83, 1.23 ± 0.69’ for the maxillary laterals; ‘97.64 ± 8.26, 2.98 ± 1.48, 3.46 ± 1.45’ for the mandibular centrals; and ‘95.98 ± 6.80, 3.29 ± 1.72, and 2.73 ± 1.15’ for the mandibular laterals. BLI was greater in the maxilla compared to the mandible and in the lateral incisors compared to centrals (*P* < 0.05). BHd was greater (more deficient) in the mandible (*P* = 0.000). Age, sex, or side were not associated with BLI (*P* > 0.05). Age, sex, side, or incisor types were not associated with BHd (*P* > 0.05). LHd was greater in the mandible, older individuals, and centrals (*P* < 0.05). There were some significant but weak correlations between BLI with BHd and especially LHd (*P* < 0.05).

**Conclusion:**

In the maxilla, but not in the mandible, incisors’ BLI may determine LHd. Maxillary incisors may have greater BLIs as well as greater buccal and lingual alveolar bone heights compared to mandibular incisors. BLI might be greater in the laterals compared to the centrals in both jaws combined.

## Introduction

Different periodontal parameters can affect the orthodontic treatment outcome. These include the morphological features of the alveolar crest and the marginal gingiva and periodontium, among others [1]. Concerning the alveolar crest height, the distance between the cementoenamel junction (CEJ) and the bone crest, which is composed of the junctional epithelium and connective tissue, is considered an important biological parameter [2]. An increased distance between the CEJ and the bone crest may be indicative of the alveolar bone dehiscence. This dehiscence may be more common after non-extraction orthodontic treatment, and can be more visible in buccal bones of the mandibular central incisors followed by bones lingual to them [2]. Also, the size and location of teeth and the alveolar bone width can affect the occurrence and degree of dehiscence [2]. Furthermore, biological factors related to the supporting bone (such as its quality and thickness) may be closely associated with anatomical dental parameters such as tooth inclination and tooth proclination [3–7]. The sum of these factors often determines the degree of potential adverse effects such as gingival recession, dehiscence, fenestration, and even external root resorption following orthodontic treatment [6, 8].

As stated above, the buccolingual inclination of incisors is a major clinical determinant of treatment success with numerous implications [3–7]. It influences numerous clinical factors such as the appearance of the patient’s profile, the well-being of the supporting soft tissue and hard tissue (i.e., vertical bone loss caused by mandibular incisor proclination), and the long-term stability of orthodontic treatment results [6]. Following orthodontic treatment, the teeth may undergo some positional changes in the alveolar sockets. The magnitude of these changes depends on the extent of orthodontic tooth movement as well as the primary morphology of the alveolar bone [6]. Therefore, the knowledge of the effect of positional changes of mandibular or maxillary incisors on the surrounding alveolar bone is imperative for orthodontic treatment planning [6]. Moreover, the anteroposterior position of mandibular or maxillary incisors can affect the fullness and position of the lips and the stability of the overbite. Thus, in order to achieve optimal esthetics and function, the knowledge of the buccolingual inclination and its affecting factors is crucial [9]. Furthermore, limited laboratory evidence suggests that the proclination of mandibular incisors can even lead to vertical bone loss [6]. Nevertheless, there is no study in this regard either on the maxilla or on humans in any jaw.

In addition to the abovementioned factors, the orthodontic treatment itself can as well cause alveolar bone loss and gingival recession [10]. Alveolar bone loss occurs more commonly in the marginal bone area because the majority of orthodontic movements are controlled tipping movements, and the retraction forces applied to incisors are often concentrated in the alveolar crest area, resulting in greater stress accumulation in the marginal region [11]. Therefore, it is necessary to scrutinize the morphology of buccal and lingual bone plates as well as tooth inclinations prior to orthodontic treatment to design an appropriate treatment plan and minimize the risk of complications such as dehiscence [2].

Very few studies have examined the associations between the incisors’ buccolingual inclinations with the vertical height of some surrounding bones in one jaw only [6, 12–14]. Nevertheless, no study exists on both jaws combined (and comparing them). Moreover, no study exists on the correlations between the incisors’ buccolingual inclinations with buccal or lingual bone heights in an epidemiologic sample. Moreover, no previous research has investigated the height of the buccal and lingual cortical plates of the maxilla and mandible at the same time. As another gap, no study has used high-resolution samples, which may improve the accuracy of measurements. Due to the importance of the incisors’ buccolingual inclination and its affecting factors, and since there is no human study on the association between the buccolingual inclination of mandibular or maxillary incisors with the heights of the buccal or lingual bone plates (which are of significant clinical importance, as stated above), this study was conducted. Its aims were (1) to assess the buccolingual inclinations (BLI) of maxillary and mandibular central and lateral incisors together with the vertical distances between their cementoenamel junctions (CEJs) with the buccal and lingual bone plates (called in this study as the buccal bone height deficiency (BHd) and lingual bone height deficiency (LHd)) using high-resolution cone-beam computed tomography (CBCT), and then (2) to analyze potential associations across them in different jaws or incisors and also (3) to examine the associations between the jaws, sexes, ages, or other factors with each of these anatomical variables. From a clinical standpoint, the use of the present study was to test whether incisor inclination has a relationship with alveolar crest height. If there was a significant relationship at all points, this study would help the orthodontist to be able to predict the limits of the alveolar crest after orthodontic treatments. The null hypotheses were the absence of any associations between BLI, BHd, and LHd, as well as demographics of the cases in the mandible, the maxilla, or both.

## Materials and methods

This 2-phase epidemiological and analytical study was conducted as two research projects (DDS theses) on 248 incisors of 62 CBCT scans of 62 patients (30 CBCT scans of the maxilla and 32 CBCT scans of the mandible). A total of 248 incisors including 124 central and 124 lateral incisors were evaluated. The study was carried out in the Oral and Maxillofacial Radiology Department of Ahvaz Jundishapur University of Medical Sciences in 2020–2021. The CBCTs obtained in this study had been all taken merely for therapeutic purposes (and not at all for any research goals). No X-ray was emitted to any individual because of this study. All radiographs were retrieved from the archives of the Radiology Department of this university and used anonymously. Therefore, no harm was identified in this research, and the ethics of this study were approved as two projects (two DDS theses) by the Ethics Committee of Ahvaz Jundishapur University of Medical Sciences following the Helsinki Declaration (ethics numbers: IR.AJUMS.REC.1399.316 and IR.AJUMS.REC.1399.317). Since this study was performed on retrospectively taken anonymized human data, the need for informed consent to participate was waived by the Institutional Review Board of the Ahvaz Jundishapur University of Medical Sciences, Ahvaz, Iran.

### Sample size

The sample size was calculated as 30 cases, based on the parameters obtained from a recent study [6], assuming 90% study power, β = 0.1, α = 0.05, and 95% confidence intervals, and using the following formula:

$$\text{n}=\frac{{\left({\text{z}}_{1-\frac{{{\upalpha }}}{2}}+{\text{z}}_{1-{\upbeta }}\right)}^{2}\left({\text{s}}_{1}^{2}+{\text{s}}_{2}^{2}\right)}{{\left(\stackrel{-}{{\text{x}}_{1}}-{\stackrel{-}{\text{x}}}_{2}\right)}^{2}}$$

To increase the power even more, the above sample size (of 30 CBCTs) was doubled-up, totaling 248 incisors in 62 CBCTs of 62 patients (32 maxillae and 30 mandibles of 62 patients).

### Eligibility criteria and the sample

The patients had to take a pretreatment CBCT for implant placement or the presence of a jaw lesion. However, the lesion should have not been in the area that was related to our study, otherwise, the patient would be excluded.

The CBCT scans are available for verification. As the sampling process, the archive was evaluated and every patient who met the inclusion criteria was enrolled. As soon as the required number of cases was reached, the sampling process was stopped. All included CBCT scans were high-resolution pre-treatment scans taken with a NewTom CBCT scanner (VGI, Verona, Italy) with an 8 × 8 cm^2^ field of view, 1.4 mA amperage, 110 kV voltage, 0.125 mm voxel size (high resolution), and 5.4 s time. The NNT Viewer software version 8.0 (VGI, Verona, Italy) was used for the assessment of CBCT scans and for measuring the variables.

Excluded were any pre-treatment CBCT scans of dentitions without incisors, with supernumerary teeth in the anterior segment, with any anterior root resorption or periapical lesions, with any restored, crowned, or endodontically treated incisors, or with moderate to severe crowding. Patients with a history of orthodontic treatment were excluded. Also, patients with periodontitis in their recorded files were excluded.

Each patient provided either a maxilla or a mandible, but not both; therefore, there was no overlap between the patients in the jaw groups. This was done because proper CBCTs with one jaw (that had been retrospectively taken for treatment purposes) were much more available.

### Anatomical assessments

**Fig. 1:**
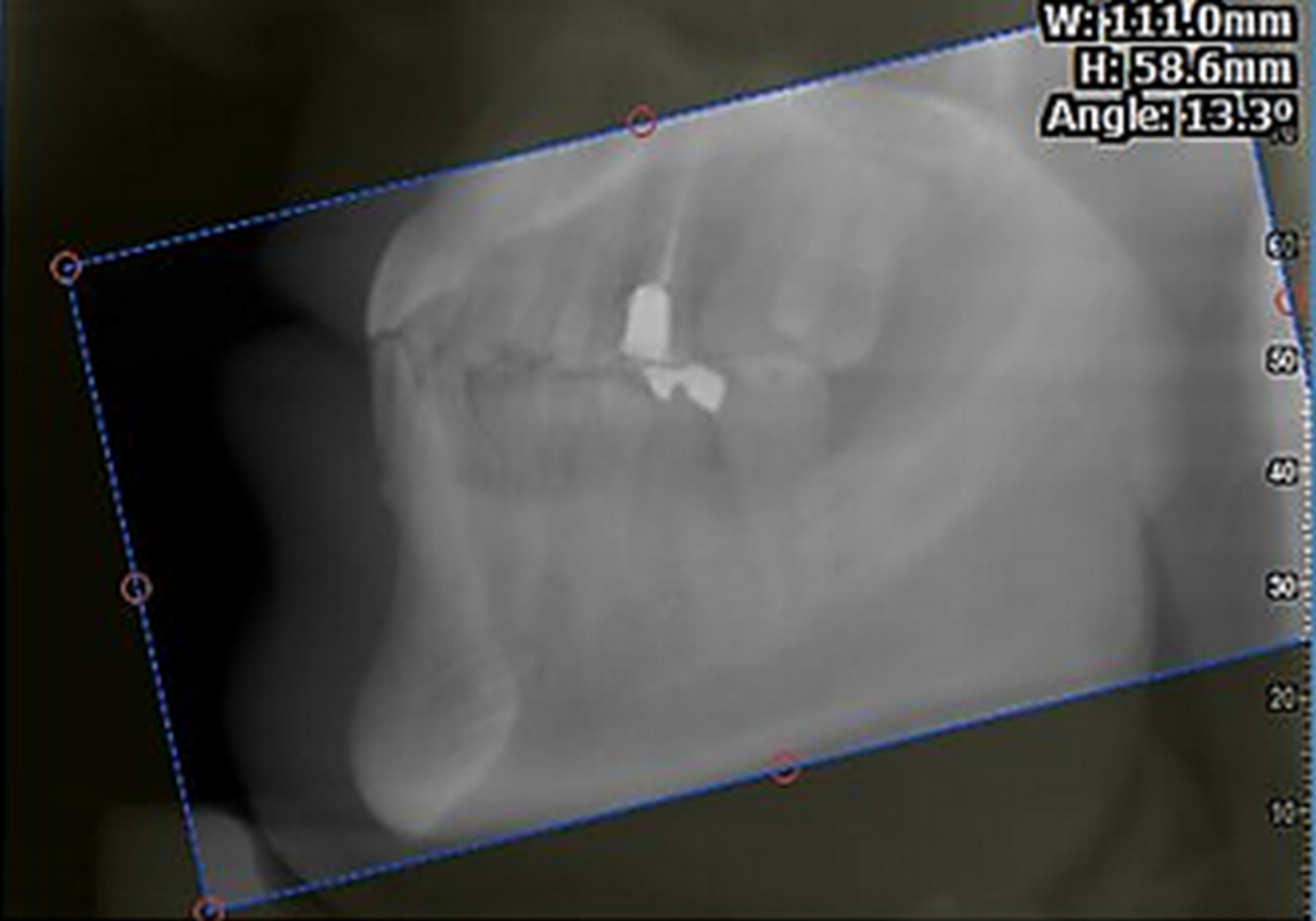
Paralleling the mandibular plane with the horizontal line

First, a lateral cephalograph was constructed from each CBCT. An accurate technique was adopted to draw the maxillary and mandibular planes because the assessment of dental inclination requires the measurement of the angle formed between the maxillary and mandibular planes and the longitudinal axis of the respective tooth. On lateral cephalographs, the palatal plane of the maxilla is defined as the line connecting the anterior nasal spine (which appears as a point on the superior or inferior contour of the nasal spine where it has 3 mm thickness) to the posterior nasal spine (at the tip of the posterior spine of the palate where soft and hard palates merge). The mandibular plane is defined as the line connecting the gonion (the midpoint of the contour connecting the mandibular body and the ramus) to the gnathion (the midpoint of the inferior border of the mandibular symphysis). However, a noteworthy issue is that in CBCT assessment, a line cannot be generalized to the entire 3D space. On the other hand, the landmarks gonion, gnathion, anterior nasal spine, and posterior nasal spine are not visible on the sagittal views of the respective incisor tooth. To solve this problem, high-resolution CBCT volumes were used. The palatal plane and the anatomical mandibular plane were adjusted parallel to the horizon such that in all images, the horizontal line indicated the maxillary or mandibular plane (Fig. 1).

**Fig. 2:**
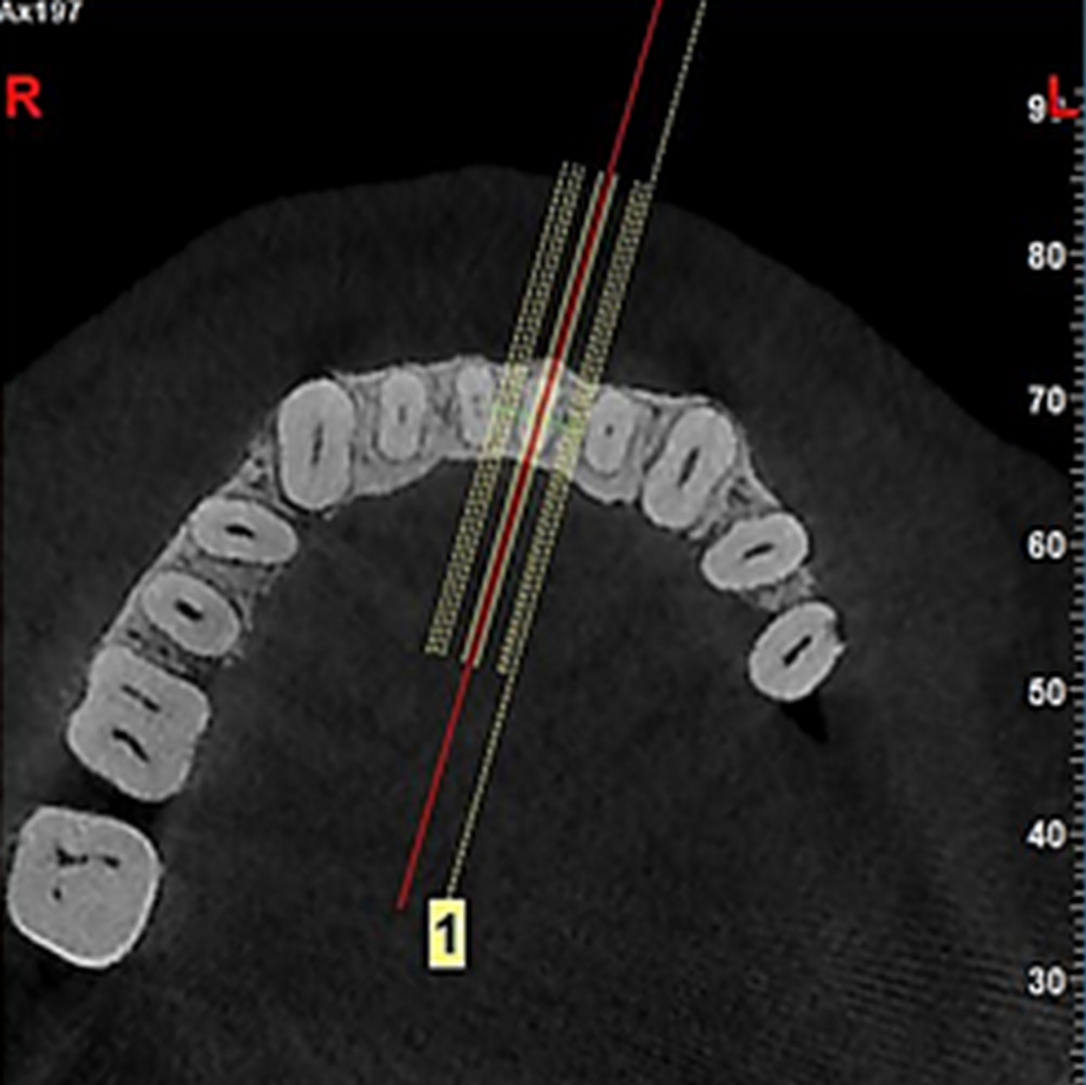
Axial view: Selecting the most appropriate section for the measurements in the sagittal plane

Afterward, to find the best section for the evaluation of incisors, the most prominent points in the buccal and lingual sides of each incisor were identified by a trained dental student on the cervical part of each tooth in the axial view, and connected with a line. This line indicated the sagittal plane, i.e., the most appropriate section for the evaluation of BLI of the tooth and marginal bone height (Fig. 2).

Next, a separate file was allocated to each incisor and coded to ensure a blind examination. For this purpose, all 248 CBCT scans of incisors were mixed, and randomly coded 001 to 248 through a simple randomization method. Then, the patients’ names were eliminated from the images to prevent possible bias by the radiologist and orthodontist and ensure a blind assessment.

**Fig. 3:**
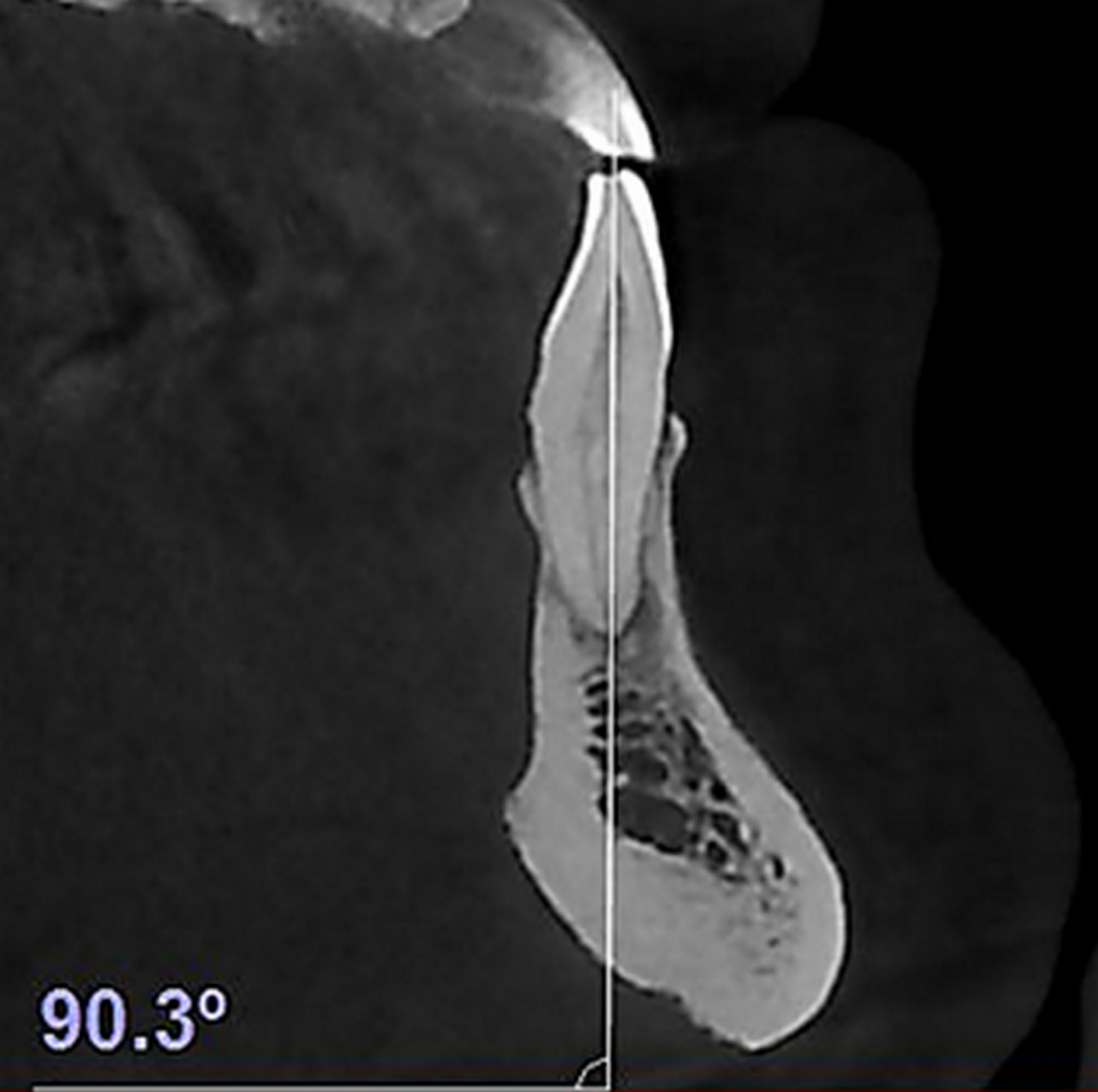
Sagittal plane: Measuring dental inclination

Subsequently, an orthodontist drew the longitudinal axis of each tooth by connecting the incisal edge to the apex. The buccolingual inclination (BLI) of each tooth was estimated by measuring the angle formed between the longitudinal axis and palatal plane in the maxilla, and the longitudinal axis and mandibular plane in the mandible (Fig. 3).

**Fig. 4:**
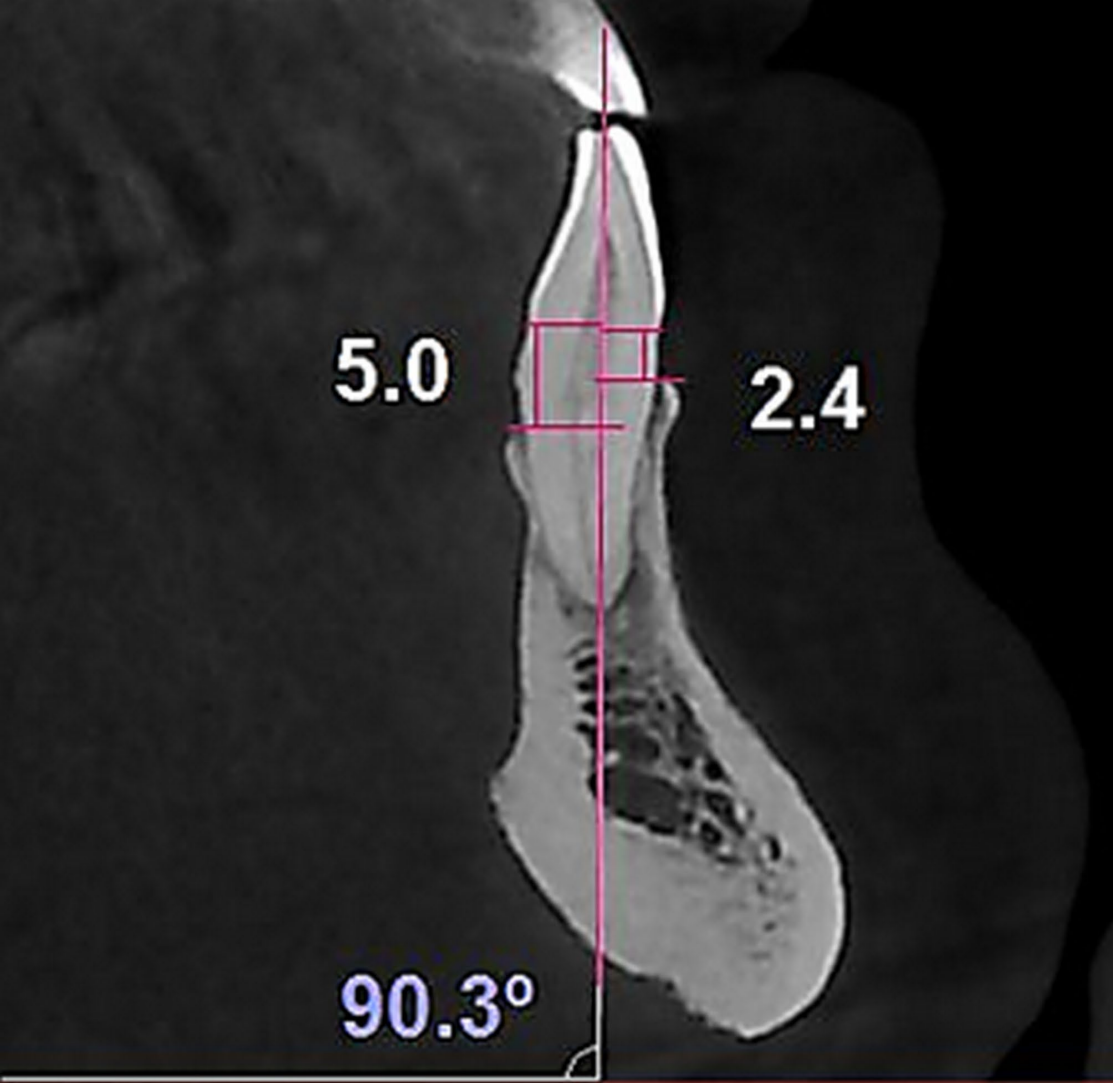
Sagittal view: Measuring the alveolar crest height on the buccal and lingual plates

To measure the buccal heigh deficiency (BHd) and the lingual heigh deficiency (LHd), a radiologist blinded to the BLI of the teeth drew a line from the CEJ in the buccal and lingual sides, perpendicular to the longitudinal axis of the tooth. Finally, a line was drawn from the bone crest in the buccal and lingual sides perpendicular to the longitudinal axis. The distance between the drawn lines and reference lines was recorded as the BHd and LHd in millimeters (Fig. 4).

### Intraobserver agreement

After 1 month, 54 teeth were randomly selected to re-measure the BLI of the teeth and BHd and LHd in order to calculate the intra-observer agreement. Reassessment of their scans revealed excellent or perfect agreements between the primary and secondary measurements (i.e., all > 95%).

### Statistical analysis

Descriptive statistics and 95% confidence intervals (CIs) were calculated. The data normality was examined and passed using a Shapiro-Wilk test and also through examining histograms and q-q plots. A Mixed-Model Linear Multiple Regression analysis was used to simultaneously assess the effects of all 5 independent variables as well as their two-way interactions on each of the 3 dependent variables “buccolingual inclination (º), buccal height deficiency (mm), and lingual height deficiency (mm)”. The 5 independent variables were: jaw [maxilla versus mandible], sex, incisor type [central versus lateral], mouth side [left versus right], and patient’s age (as a continuous variable, in years). The repeated measurements were considered the paired data pertaining to the 4 incisors of each subject. Each model was optimized manually. The Pearson correlation coefficient was calculated to confirm the presence of a correlation across the traits. The software in use was SPSS version 25 (IBM, Armonk, NY, USA). The level of significance was set at 0.05.

## Results

There was no missing data. The included maxillae belonged to 13 men and 17 women; the mandibles were from 13 men and 19 women. The sex distributions of the jaws were similar (Fisher, *P* = 1.0). The mean age of the 62 patients was 40.84 ± 11.79 years (minimum: 23, maximum: 68). The mean ages of 30 patients with maxillae and 32 patients with mandibles were respectively 39.30 ± 10.22 years (minimum: 25, maximum: 65) and 42.28 ± 13.10 years (minimum: 23, maximum: 68). The ages of patients with maxilla versus mandible were not significantly different (t-test, *P* = 0.324). The mean ages of 26 males and 36 females were respectively 43.85 ± 10.37 years (minimum: 29, maximum: 65) and 38.67 ± 12.41 years (minimum: 23, maximum: 68). The ages of men and women were not significantly different (t-test, *P* = 0.088). In the maxilla group, the mean ages of 13 males and 17 females were respectively 40.77 ± 10.04 years and 38.18 ± 10.51 years (t-test, *P* = 0.501). In the mandible group, the mean ages of 13 males and 19 females were respectively 46.92 ± 10.13 years and 39.11 ± 14.17 years (t-test, *P* = 0.098).

### Results of the mixed-model linear multiple regression

Table 1 shows the descriptive statistics and 95% CIs for the 3 dependent variables in different subgroups.

**Table 1:** Descriptive statistics and 95% CIs for the examined anatomical parameters in all subgroups

Jaw	Incisor	Parameter	Sex	N	Mean	SD	95% CI		Min	Max
**Maxilla**	**Central**	**Buccolingual Inclination (º)**	**Female**	34	108.00	6.06	105.89	110.12	99.20	118.00
			**Male**	26	105.20	2.70	104.10	106.29	100.50	109.00
			**Total**	60	106.79	5.06	105.48	108.09	99.20	118.00
		**Buccal Bone Height deficiency (mm)**	**Female**	34	1.64	0.62	1.42	1.86	0.80	3.20
			**Male**	26	2.33	1.15	1.87	2.80	0.90	5.80
			**Total**	60	1.94	0.95	1.70	2.18	0.80	5.80
		**Lingual Bone Height deficiency (mm)**	**Female**	34	1.57	0.89	1.26	1.88	0.60	4.00
			**Male**	26	1.41	0.55	1.19	1.63	0.60	2.90
			**Total**	60	1.50	0.76	1.30	1.70	0.60	4.00
	**Lateral**	**Buccolingual Inclination (º)**	**Female**	34	111.28	6.27	109.09	113.46	96.50	123.60
			**Male**	26	109.62	5.53	107.39	111.86	99.80	122.50
			**Total**	60	110.56	5.97	109.02	112.10	96.50	123.60
		**Buccal Bone Height deficiency (mm)**	**Female**	34	1.85	0.84	1.56	2.15	0.80	4.60
			**Male**	26	1.76	0.84	1.42	2.10	0.80	4.40
			**Total**	60	1.81	0.83	1.60	2.03	0.80	4.60
		**Lingual Bone Height deficiency (mm)**	**Female**	34	1.18	0.66	0.95	1.41	0.00	3.20
			**Male**	26	1.29	0.74	0.99	1.59	0.40	3.30
			**Total**	60	1.23	0.69	1.05	1.41	0.00	3.30
**Mandible**	**Central**	**Buccolingual Inclination (º)**	**Female**	38	96.65	8.63	93.81	99.48	77.20	112.30
			**Male**	26	99.08	7.64	96.00	102.17	83.80	112.40
			**Total**	64	97.64	8.26	95.57	99.70	77.20	112.40
		**Buccal Bone Height deficiency (mm)**	**Female**	38	2.97	1.64	2.43	3.51	0.50	9.30
			**Male**	26	3.00	1.23	2.50	3.49	1.10	5.80
			**Total**	64	2.98	1.48	2.61	3.35	0.50	9.30
		**Lingual Bone Height deficiency (mm)**	**Female**	38	3.36	1.45	2.88	3.84	1.50	7.30
			**Male**	26	3.61	1.48	3.02	4.21	1.20	6.90
			**Total**	64	3.46	1.45	3.10	3.83	1.20	7.30
	**Lateral**	**Buccolingual Inclination (º)**	**Female**	38	95.90	7.16	93.55	98.25	79.70	112.80
			**Male**	26	96.10	6.37	93.52	98.67	84.40	107.90
			**Total**	64	95.98	6.80	94.28	97.68	79.70	112.80
		**Buccal Bone Height deficiency (mm)**	**Female**	38	3.58	2.09	2.89	4.26	1.20	10.20
			**Male**	26	2.88	0.86	2.53	3.22	1.40	4.40
			**Total**	64	3.29	1.72	2.86	3.72	1.20	10.20
		**Lingual Bone Height deficiency (mm)**	**Female**	38	2.56	1.16	2.17	2.94	1.20	6.80
			**Male**	26	2.98	1.10	2.53	3.43	1.50	5.00
			**Total**	64	2.73	1.15	2.44	3.01	1.20	6.80

**SD**, standard deviation; **CI**, confidence interval; **Min**, minimum; **Max**, maximum

**Fig. 5:**
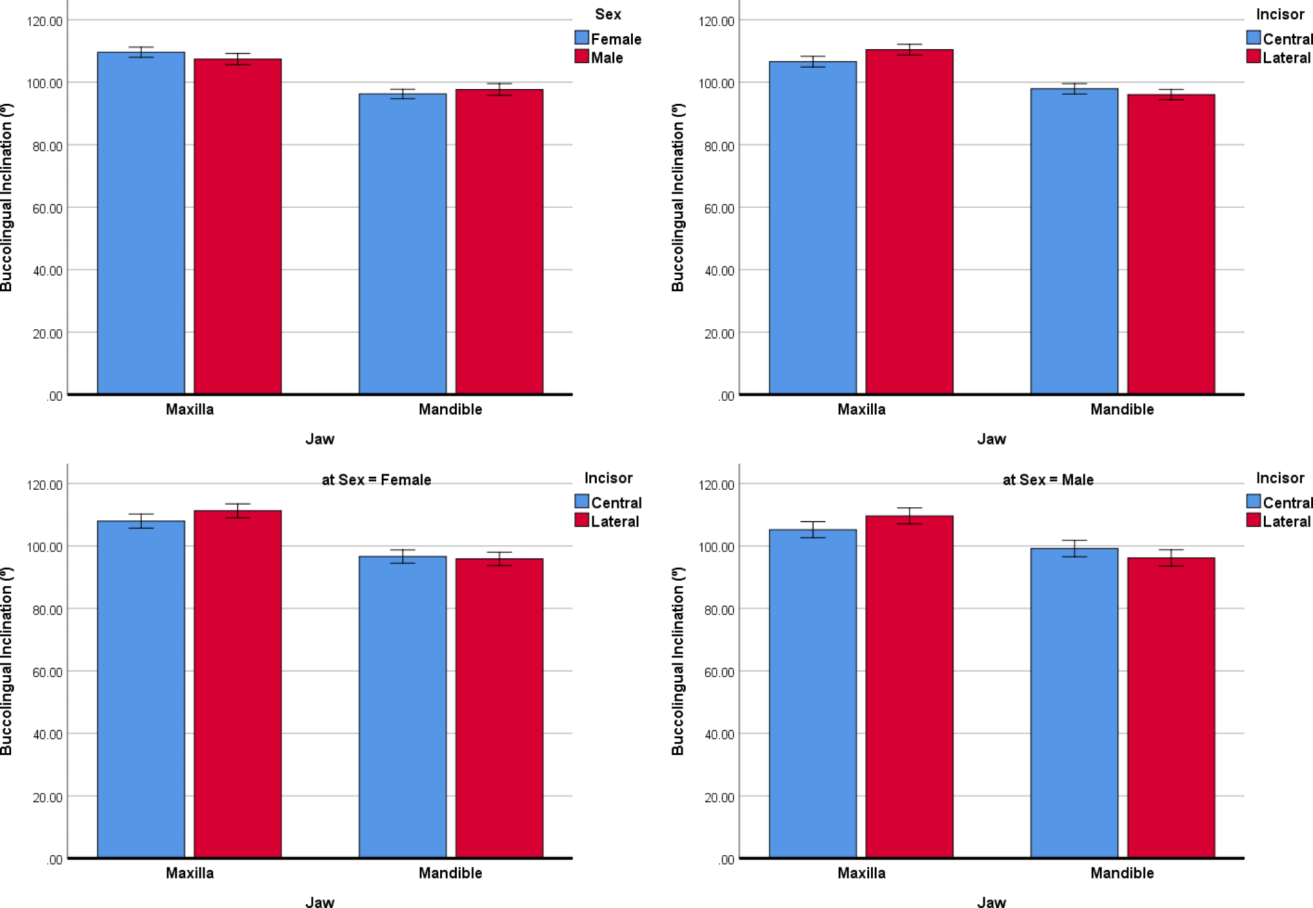
Estimated marginal means (and 95% CIs) for buccolingual inclinations (º) in different subgroups

BLI: The mixed regression showed that only the jaws and incisors were associated with BLI: the buccolingual inclinations of the teeth differed between the mandible versus the maxilla (greater in the maxilla, *P* < 0.00005, Fig. 5); it was greater in the laterals compared to the centrals (*P* = 0.0005). None of the other independent variables ‘age (*P* = 0.881), sex (*P* = 0.314, in the first model), or side (*P* = 0.195, in the first model)’ were associated with the buccolingual inclination of the teeth. The interactions between incisor types with jaws became significant (*P* < 0.00005).

**Fig. 6:**
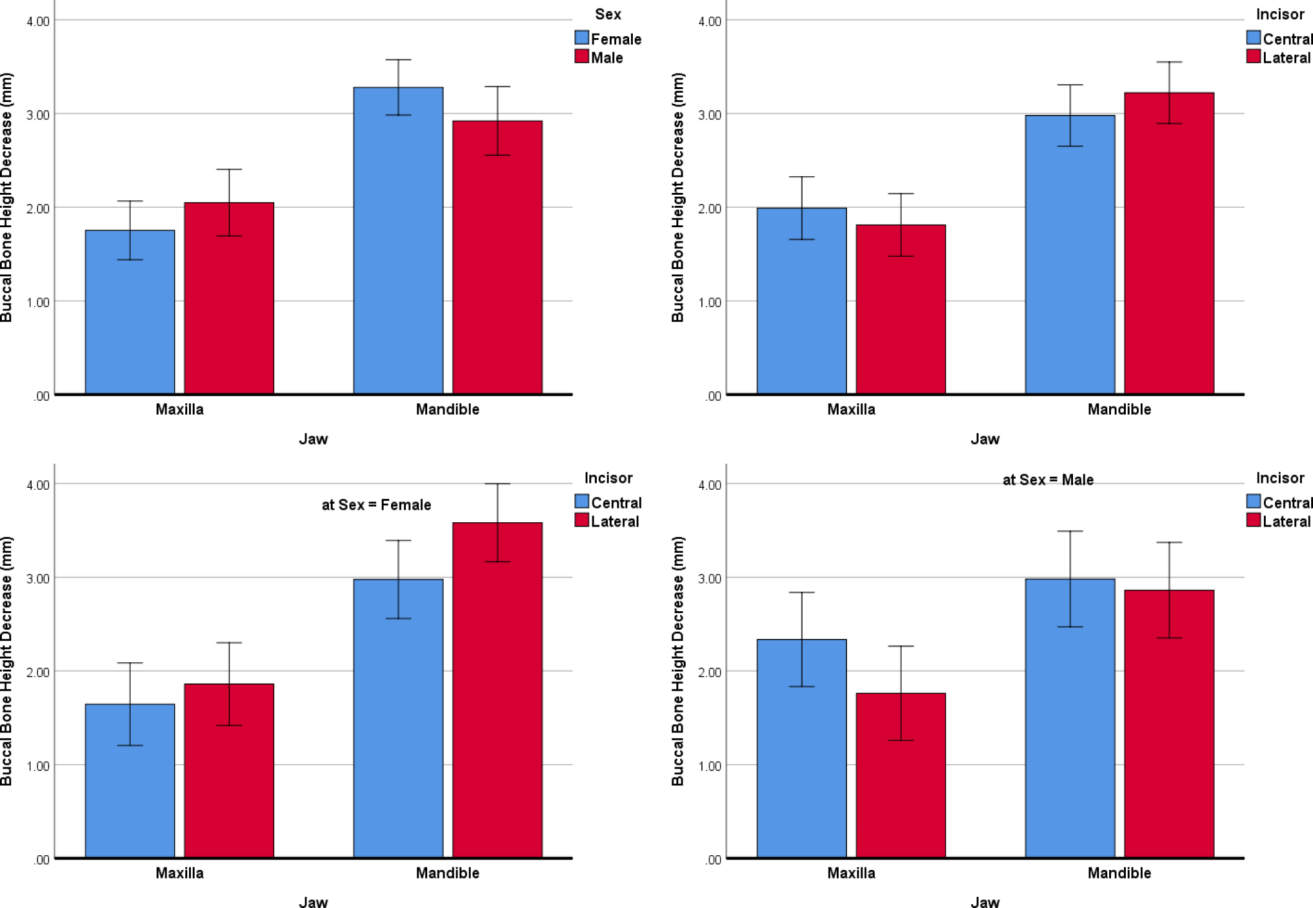
Estimated marginal means (and 95% CIs) for deficiencies in the buccal bone heights (mm) in different subgroups

BHd: After optimizing the mixed model by removing the nonsignificant variable side (*P* = 0.913) and age (*P* = 0.981), only the variable ‘jaw’ turned out to be associated with the deficiency in the buccal bone height (more deficient in the mandible, *P* < 0.00005, Fig. 6). None of the other independent variables ‘sex (*P* = 0.946), or incisor type (*P* = 0.805)’ were associated with the deficiency in the buccal bone height. The interactions of Sex by Jaw (*P* = 0.211) and Jaw × Incisor type (*P* = 0.100) were not significant. However, the interaction of Sex by Incisor type was significant (*P* = 0.004).

**Fig. 7:**
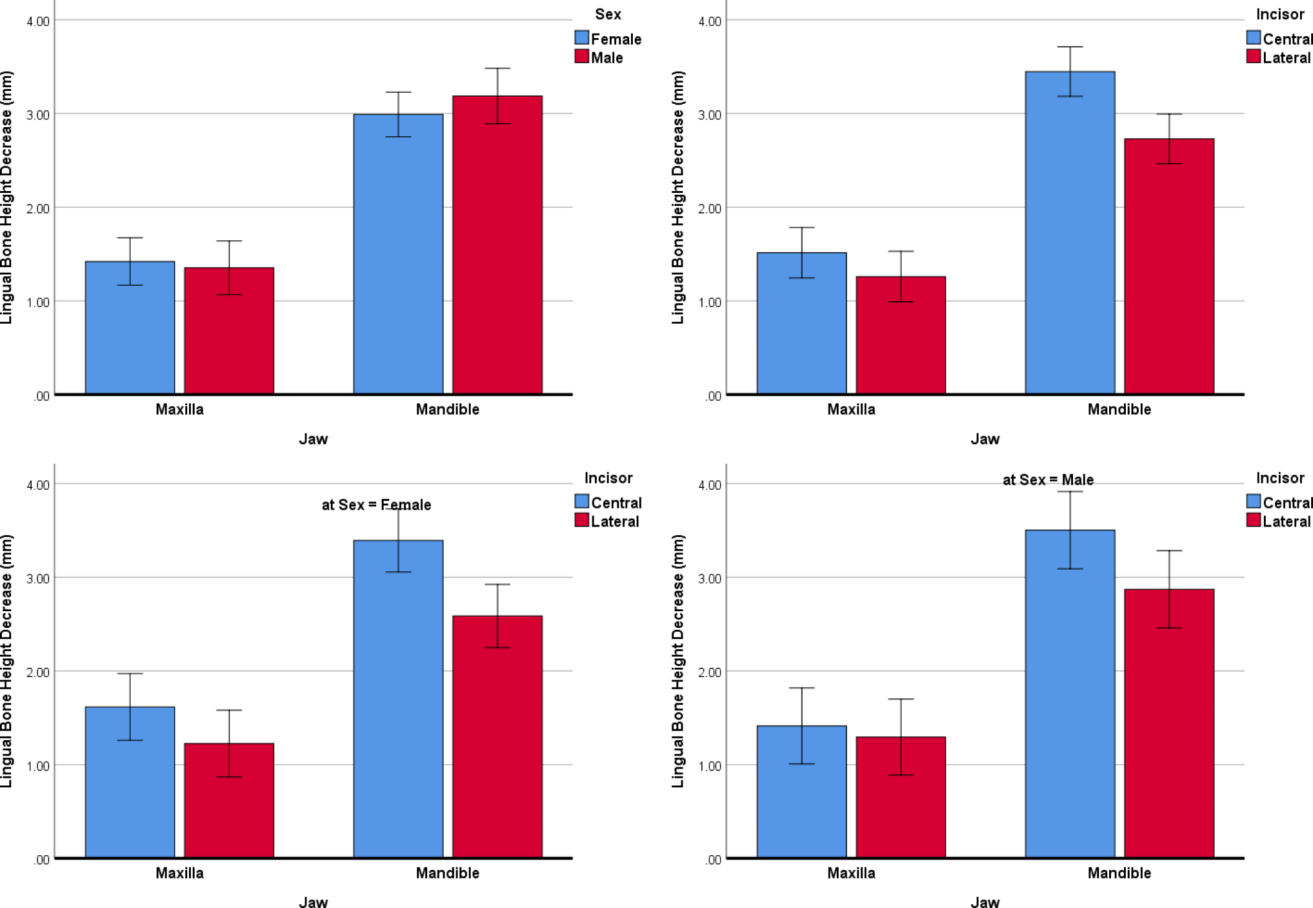
Estimated marginal means (and 95% CIs) for deficiencies in the lingual bone heights (mm) in different subgroups

**Fig. 8:**
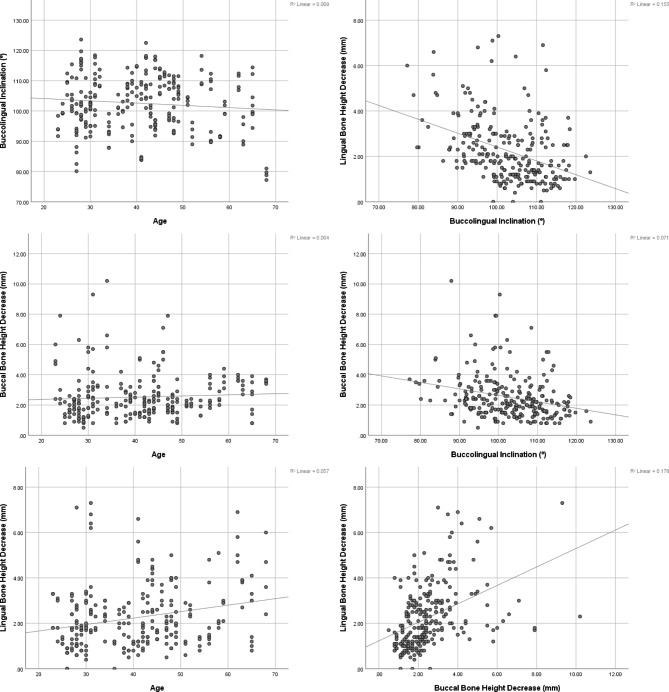
Scatterplots illustrating correlations between the continuous variables. The left column shows correlations between age with each of the three anatomical variables when both incisors and also both jaws are combined. The right column shows correlations between anatomical variables (both incisors and both jaws are combined)

LHd: According to the mixed regression, the deficiency in the lingual bone height was associated with the jaws (more deficient in the mandible, Table 2; Fig. 7), age (more deficient in older patients, Table 2; Fig. 8), and incisors (more deficient in the centrals, Table 2; Fig. 7). Sex and side were not associated with the deficiency in lingual bone height (Table 2). None of the interactions was significant (Table 2), that is the effect of each of the independent variables on LHd was not influenced significantly by the effect of another predictor.

**Table 2:** The results of the mixed-model multiple linear regression, identifying the predictors of the lingual bone height deficiency

Model	Source	F	*P*
**1**	**Intercept**	4.574	0.032481
	**Sex**	0.014	0.905597
	**Jaw**	6.225	**0.013293**
	**Incisor type**	0.104	0.746816
	**Side**	0.530	0.467260
	**Age**	7.203	**0.007803**
	**Sex by Jaw**	0.526	0.468879
	**Sex by Incisor**	1.191	0.276303
	**Sex by Side**	0.025	0.874841
	**Sex by Age**	0.052	0.818984
	**Jaw by Incisor**	2.230	0.136734
	**Jaw by Side**	0.000	0.994273
	**Jaw by Age**	0.515	0.473560
	**Incisor by Side**	0.345	0.557244
	**Incisor by Age**	1.761	0.185778
	**Side by Age**	0.417	0.519098
**5**	**Intercept**	12.718	0.000726
	**Jaw**	56.490	**< 0.000001**
	**Incisor**	33.106	**< 0.000001**
	**Age**	4.137	**0.046455**

Intercept denotes the mean value of the dependent variable (LHd) when the predictors are zero. Significant *P* values in bold font

### Correlations

The Pearson correlation coefficient showed that age was positively correlated with the deficiency in the lingual bone height, in the centrals of the maxilla and mandible (Table 3) which also made the overall correlation significant as well (Table 4; Fig. 8). There was a significant correlation between BHd and LHd in mandibular central incisors (Table 3). There were also some significant but weak correlations between BLI with BHd and LHd (Tables 3 and 4).

**Table 3:** Correlations among the continuous variables in each incisor of each jaw

Incisor	Variables	Statistic	Maxilla (n = 60)			Mandible (n = 64)		
			BLI	BHd	LHd	BLI	BHd	LHd
**Central**	**Age**	**R**	0.0460	-0.0089	0.3166	-0.0415	-0.0055	0.2414
		***P***	0.7271	0.9464	**0.0137**	0.7448	0.9656	**0.0546**
	**BLI**	**R**		-0.0674	0.3660		0.0028	-0.1426
		***P***		0.6086	**0.0040**		0.9826	0.2610
	**BHd**	**R**	-0.0674		0.1208	0.0028		0.4348
		***P***	0.6086		0.3579	0.9826		**0.0003**
	**LHd**	**R**	0.3660	0.1208		-0.1426	0.4348	
		***P***	**0.0040**	0.3579		0.2610	**0.0003**	
**Lateral**	**Age**	**R**	0.0519	0.0071	0.0191	-0.0696	0.0218	0.2213
		***P***	0.6940	0.9568	0.8848	0.5850	0.8643	0.0788
	**BLI**	**R**		-0.0156	0.2768		0.1597	-0.0049
		***P***		0.9057	**0.0323**		0.2074	0.9691
	**BHd**	**R**	-0.0156		0.3900	0.1597		0.0459
		***P***	0.9057		**0.0021**	0.2074		0.7188
	**LHd**	**R**	0.2768	0.3900		-0.0049	0.0459	
		***P***	**0.0323**	**0.0021**		0.9691	0.7188	

Significant *P* values in bold font. R = Pearson correlation coefficient

**Table 4:** Correlations among the continuous variables in both incisors combined

Variables	Statistic	Both jaws (n = 248)			Maxilla (n = 120)			Mandible (n = 128)		
		BLI	BHd	LHd	BLI	BHd	LHd	BLI	BHd	LHd
**Age**	**R**	-0.0972	0.0607	0.2385	0.0463	-0.0014	0.1711	-0.0536	0.0091	0.2222
	***P***	0.1270	0.3409	**0.0001**	0.6153	0.9882	0.0616	0.5481	0.9187	**0.0117**
**BLI**	**R**		-0.2662	-0.3933		-0.0615	0.2340		0.0669	-0.0547
	***P***		**< 0.00005**	**< 0.00005**		0.5044	**0.0101**		0.4531	0.5396
**BHd**	**R**	-0.2662		0.4223	-0.0615		0.2494	0.0669		0.2071
	***P***	**< 0.00005**		**< 0.00005**	0.5044		**0.0060**	0.4531		**0.0190**
**LHd**	**R**	-0.3933	0.4223		0.2340	0.2494		-0.0547	0.2071	
	***P***	**< 0.00005**	**< 0.00005**		**0.0101**	**0.0060**		0.5396	**0.0190**	

Significant *P* values in bold font. R = Pearson correlation coefficient

When assessing the laterals in the mandible, no significant correlation was observed among BLI, BHd, or LHd (Table 3). However, maxillary laterals showed a weak yet significant correlation between BLI and LHd (Table 3). Age was correlated with lingual bone height deficiency (but not BHd, Table 3).

After combining both incisors in each jaw separately, it was found that BLI was correlated only to LHd in the maxilla (Table 4). However, after also combining both jaws, it was found that BLI was correlated with both BHd and LHd (Table 4; Fig. 8). BHd was correlated with LHd in each jaw separately, and also in both jaws combined (Table 4; Fig. 8).

## Discussion

The findings of this study indicated that the maxillary incisors had greater buccolingual inclinations and alveolar bone heights compared to the mandibular incisors. Moreover, BLI was the laterals had greater BLIs than the centrals. BLI was not associated with the side of mouth as well as patients’ sex or age. Similarly, BHd was not associated with age, sex, side, or incisor types. Aging decreases lingual alveolar bone height. Central incisors had smaller lingual bone heights than the laterals. LHd was not associated with patients’ sex. Deficiencies in buccal and lingual bone heights were correlated with each other in each jaw. There correlations between BLI with BHd and especially LHd were significant but weak.

No similar studies were found on this topic to compare our results with. Thus, a few relatively similar studies were discussed instead. Our results showed that in the maxilla, BLI of incisors was significantly correlated with LHd. Also, BHd and LHd were significantly correlated in a direct way. Nonetheless, BLI of maxillary incisors and BH were not significantly correlated. Also, BLI of mandibular incisors was not correlated with either BHd or LHd. However, BH and LH had a direct correlation with each other in the mandible. Khyati et al. [6] found no significant correlation between the marginal bone height in different skeletal patterns and tooth inclinations, which was in agreement with the present findings. Also, Kamak et al. [12] showed that the increase in clinical crown height (as an indicator of gingival recession and loss of periodontal structure) was not significant in any group, which was in line with the present findings. Bonta et al. [13] found no significant correlation between the buccal position of the teeth and the distance between the CEJ to the osseous zenith. Their results were in agreement with the present findings. Although the results of previous studies on this topic were in agreement with the present results, some differences existed in the obtained statistical values, which could be due to numerous methodological differences as well as variations in the characteristics of study populations. Moreover, it should be noted that the previous studies were not similar to our study and had different goals. In addition, high-resolution CBCT scans were used in the present study to increase the accuracy of measurements, unlike the previous studies on this topic; this again can contribute to the differences.

We found that the incisors were inclined more labially in the maxilla compared to the mandible. The distance between the CEJ with the buccal crest was greater in the mandible compared to the maxilla. None of these anatomical parameters was affected by the age or sex of the patient or by the type of incisor. The distance between the CEJ with the lingual bone height was greater in the mandible than in the maxilla; it was greater in the central incisors compared to lateral ones; and it became greater by age. None of these 3 anatomical variables was affected by patients’ sexes. We could not find studies evaluating any of these items in order to compare our results, except for the effect of age. Of course, some cortical bone loss can be expected as individuals age [15, 16]. Nevertheless, age did not affect the buccal bone height deficiency. The explanation of this finding needs future studies, but it may be contemplated that perhaps the form and trabeculation of the buccal tables make its height more stable.

Although a previous study reported an underestimation of bone height in the buccal plate and an overestimation of the presence of dehiscence and fenestration by CBCT [14], some others showed no significant difference between CBCT findings and the gold standard (direct assessment of human skulls and living patients) in the examination of the alveolar bone height and thickness [17]. Three-dimensional analysis of the alveolar bone using CBCT is significantly superior to the older radiographic modalities such as plain radiography because it better visualizes the actual three-dimensional structure of bone, and minimizes the problems such as distortion and superimposition of adjacent structures. The optimally high accuracy, advantages, and superiority of CBCT over conventional radiographic modalities have been well documented in the literature [11].

Although our study power was very high, still further studies with larger sample sizes on different ethnic groups would enrich the literature. A limitation of all X-ray studies is that since X-ray is dangerous to humans, researchers cannot expose any subjects to CBCT for the sake of their research. Therefore, we like all other researchers were limited to examining only available CBCTs that had been already taken retrospectively for therapeutic purposes. The same reason (i.e., the hazard of X-ray) disallows clinicians to take CBCTs with unnecessarily large fields of view, when the patient is healthy and does not have any major anatomical issues or treatment needs. Therefore, as another limitation of this study, no CBCTs were available that can simultaneously meet these 2 conditions: (1) having a very large field of view to encompass both jaws of a person simultaneously, and (2) taken from patients that could meet the eligibility criteria of this study. Hence, we were limited to use CBCTs of single jaws, and as a result no mandibles were matched with no maxillae in terms of genetics, etc. Another limitation was the lack of assessment of some more anatomical parameters. We could also evaluate the alveolar crest width and buccolingual width of the alveolar ridge. The reason for not doing this was the lack of novelty of those particular assessments. Therefore, we preferred to focus our limited resources on the evaluation of more novel aspects. In addition, occlusion can affect the buccolingual inclination of teeth as well as the prevalence of dehiscence and fenestration [5, 8]. Hence, it would be better if we could also assess the role of occlusion. Nevertheless, information pertaining to patients’ occlusions was not available within the data files, and the single-jaw CBCT volumes did not allow us to detect occlusion from CBCTs. As future directions, assessment of the prevalence of fenestration in the buccal alveolar crest of incisors with no history of orthodontic treatment and its comparison with the value after orthodontic treatment may provide valuable information regarding the morphology of buccal alveolar crest, and type and magnitude of response to orthodontic treatment.

## Conclusion

It was shown that the maxillary incisors (compared to the mandibular ones) may have greater buccolingual inclinations as well as greater buccal and lingual alveolar bone heights (indicated by their smaller deficiencies in their buccal and lingual alveolar bone heights [i.e., smaller maxillary BHd and smaller maxillary LHd] compared to the mandible). Also, BLI might be greater in the laterals compared to the centrals. Age, sex, or side were not associated with BLI. Age, sex, side, or incisor types were not associated with BHd. Aging reduces lingual alveolar bone height (indicated by greater LHd values in older people). The central incisors may have smaller lingual alveolar bone heights [or greater LHds] compared to the lateral incisors. Sex was not associated with LHd.

There were correlations among the variables. For instance, buccal and lingual bone height deficiencies were correlated with each other in each jaw. There were also some significant but weak correlations between BLI with BHd and especially LHd.

As clinical merit, the risk factors proposed for deficient buccal or lingual bone heights may be considered in treatment planning by the clinician to avoid tooth mobility or gingival recession caused by worsened alveolar defects.

## Data Availability

The data are available from the corresponding author upon request.
